# Mr. Tomes' Lectures

**Published:** 1847-10

**Authors:** 


*Mr. Tomes' Lectures.*—We commenced the publication of a course of lectures delivered by Mr. Tomes, an eminent English dentist, before the Middlesex Hospital, upon the anatomy, physiology and pathology of the teeth. We have resumed their publication in the present number, and shall continue them in our next. These lectures are ably written, and will be read, we doubt not, with interest by the subscribers of the Journal. They contain a vast amount of valuable information upon the subjects upon which they treat.—*Balt. Ed.*

